# CXCR2 signaling might have a tumor-suppressive role in patients with cholangiocarcinoma

**DOI:** 10.1371/journal.pone.0266027

**Published:** 2022-04-04

**Authors:** Yurie Yamamoto, Atsushi Sugimoto, Koji Maruo, Gen Tsujio, Tomohiro Sera, Shuhei Kushiyama, Sadaaki Nishimura, Kenji Kuroda, Shingo Togano, Shinpei Eguchi, Ryota Tanaka, Kenjiro Kimura, Ryosuke Amano, Masaichi Ohira, Masakazu Yashiro

**Affiliations:** 1 Molecular Oncology and Therapeutics, Osaka City University Graduate School of Medicine, Osaka, Japan; 2 Cancer Center for Translational Research, Osaka City University Graduate School of Medicine, Osaka, Japan; 3 Department of Gastroenterological Surgery, Osaka City University Graduate School of Medicine, Osaka, Japan; 4 Department of Hepato-Biliary-Pancreatic Surgery, Osaka City University Graduate School of Medicine, Osaka, Japan; Texas A&M University, UNITED STATES

## Abstract

**Background:**

We reported that chemokine C-X-C motif receptor 2 (CXCR2) signaling appears to play an important role in the pathogenic signaling of gastric cancer (GC), and although CXCR2 may have a role in other solid cancers, the significance of CXCR2 in cholangiocarcinoma (CCA) has not been evaluated. Herein, we determined the clinicopathologic significance of CXCL1-CXCR2 signaling in CCA.

**Materials and methods:**

Two human CCA cell lines, OCUG-1 and HuCCT1, were used. CXCR2 expression was examined by western blotting. We investigated the effects of CXCL1 on the proliferation (by MTT assay) and migration activity (by a wound-healing assay) of each cell line. Our immunohistochemical study of the cases of 178 CCA patients examined the expression levels of CXCR2 and CXCL1, and we analyzed the relationship between these expression levels and the patients’ clinicopathologic features.

**Results:**

CXCR2 was expressed on both CCA cell lines. CXCL1 significantly inhibited both the proliferative activity and migratory activity of both cell lines. CXCL1 and CXCR2 were immunohistochemically expressed in 73% and 18% of the CCA cases, respectively. The CXCL1-positive group was significantly associated with negative lymph node metastasis (p = 0.043). The CXCR2-positive group showed significantly better survival (p = 0.042, Kaplan-Meier). A multivariate logistic regression analysis revealed that CXCR2 expression (p = 0.031) and lymph node metastasis (p = 0.004) were significantly correlated with the CCA patients’ overall survival.

**Conclusion:**

CXCR2 signaling might exert a tumor-suppressive effect on CCA cells. CXCR2 might be a useful independent prognostic marker for CCA patients after surgical resection.

## Introduction

Cholangiocarcinoma (CCA) is a biliary cancer that may originate from epithelial cells in the biliary tract, and patients with CCA have had an extremely poor prognosis [1]. Most CCAs are diagnosed at an advanced stage, and there are few treatment options; the development of effective treatments is thus desired [2]. Several factors that can serve as biomarkers in CCA were recently reported [3–6], and some chemokines have been shown to play an important role in the development of CCA [1, 7]. Indeed, chemokines are known to have roles in the development of various types of cancer [8–10]. Chemokine C-X-C motif receptor 2 (CXCR2) has many ligands, such as chemokine (C-X-C motif) ligand 1 (CXCL1), CXCL2, CXCL3, CXCL5, CXCL6, CXCL7, and CXCL8 [11], and the clinical significance of CXCL1 and CXCR2 and their signaling has not been clarified.

It was reported that CXCL7 and CXCL5 are involved in the development of CCA [1, 12, 13]. CXCL5/ENA-78 (epithelial cell-derived neutrophil-activating peptide-78) was identified as a factor in the interaction between CCA cells and cancer-associated fibroblasts [13]. In addition, various chemokines have been reported to act as tumor suppressors and indicators of better prognosis in gastric cancer [14], colorectal cancer [15–17], lung adenocarcinoma [18, 19], and triple-negative breast cancer [20]. These findings might indicate that CXCR2 signaling is a target in various cancers, but the effects of this signaling on cancer cells might differ among cancer types [11]. We observed that among the members of the family of CXCR2 ligands, CXCL1, one of the ligands for CXCR2, produced by cancer cells might play an important role among CXCR2 ligands family in gastric cancer [21]. These findings suggest that CXCL1-CXCR2 signaling may exert one or more actions in the development of CCA. We conducted the present study to examine the significance of CXCL1-CXCR2 signaling in CCA.

## Materials and methods

### Cell lines

Two human CCA cell lines, OCUG-1 and HuCCT1(RCB1960), were used. The OCUG-1 cell line was established at our laboratory [22]. The HuCCT1 line was provided by RIKEN BRC (BioResource Research Center) (Tsukuba, Japan). All cell lines in this study were authenticated by short tandem repeat (STR) profiling before distribution.

### Cell culture

The culture medium consisted of Dulbecco’s modified Eagle medium (DMEM; Wako, Osaka, Japan) with high glucose, penicillin-streptomycin solution (5 mL/500 mL D-MEM, Wako), 100 mM sodium pyruvate solution (5 mL/500 mL D-MEM, Sigma-Aldrich, St. Louis, MO), and 10% fetal bovine serum (FBS, Sigma). Cells were cultured at 37°C in 20% O_2_.

### Compounds

CXCL1 recombinant (BioLegend, San Diego, CA) was used.

### Proliferation assay

The effect of CXCL1 on the proliferation of CCA cells was determined by an MTT assay (Dojindo, Kumamoto, Japan) as follows. A total of 2.5×10^3^ cells were seeded into 96-well plates with CXCL1 at 20 ng/mL. After incubation for 72 h, MTT was added to each well. After incubation for 2 h at 37°C, the plate was measured with absorbance at 570  nm using a microtiter plate reader.

### Wound-healing assay

Both cell lines were cultured in 96-well plates (Essen ImageLock; Essen Instruments, Birmingham, UK). After the cells reached semi confluence, a wound was generated in the cell monolayer with the 96-well WoundMaker (Essen BioScience Inc., Ann Arbor, MI). Cell lines were cultured in DMEM with 3% FBS and CXCL1 at 20 ng/mL or control. Scratched fields were imaged every 12 h and monitored for 48 h with the Incucyte ZOOM Live-Cell Imaging System and software ver. 2018A (Essen Instruments).

### Western blot analysis

Cell lysates of OCUG-1 and HuCCT1 were made by standard methods. The protein concentration of each sample was measured using a Coomassie Plus Assay Kit (Thermo Fisher Scientific, Waltham, MA). Each sample was subjected to electrophoresis and transferred to a polyvinylidene difluoride membrane. The membrane was placed in phosphate-buffered saline with Tween 20 (PBS-T) solution containing each primary antibody: CXCR2 (1:2000, R&D Systems, Minneapolis, MN) or β-actin (1:5000; Sigma-Aldrich) at 4°C overnight. The blots were incubated with secondary antibody for 1 h and were detected by enhanced chemiluminescence using ECL prime (GE Health Care, Buckinghamshire, UK).

### Clinical materials

We also retrospectively analyzed the cases of 178 patients with CCA who underwent surgery at Osaka City University Hospital. CCA tissues were obtained from each patient. The pathological diagnosis and classifications were made according to the Japanese classification of CCA (6th edition). This study was approved by the Osaka City University Ethics Committee (approval no. 924). Written informed consent for this research was obtained from all of the patients.

### Immunohistochemical determination of CXCL1 and CXCR2

We conducted the present experiment based on the methods reported in our previous papers [21]. The immunohistochemical determination of the expressions of CXCL1 and CXCR2 in the CCA tissues from the patients was performed as follows. The tissue samples were deparaffinized, and slides were heated. After endogenous peroxidase activity was blocked, the samples were incubated with anti-CXCL1 antibody (1:200; Proteintech, Rosemont, IL) or anti-CXCR2 antibody (1:100; R&D Systems) for 1 h at room temperature. The samples were then incubated with biotinylated secondary antibody, followed by treatment with streptavidin-peroxidase reagent and counterstaining with Mayer’s hematoxylin.

The expression levels of CXCL1 and CXCR2 were analyzed by both the intensity of staining and the percentage of stained cancer cells at the invading tumor front. The CXCL1 and CXCR2 expression levels were evaluated as follows: the intensity scores were 0–3 (0  =  no, 1  =  weak, 2  =  moderate, 3  =  intense), and the percentage of immune-positive cells was given the scores 0–3 (CXCL1: 0  =  0%–20%, 1  =  21%–50%, 2  =  51%–80%, 3  =  81%–100%, and CXCR2: 0  =  0%, 1  =  1%–30%, 2  =  31%–70%, 3  =  71%–100%). The two scores were added to gain the final result of 0–6 points. Expression was considered positive when the CXCL1 score was ≥4 and when the CXCR2 score was ≥5. The evaluations were conducted by two double-blinded independent observers who were unaware of the patients’ clinical data and outcomes. When the observers reached different conclusions about an evaluation, it was rechecked and resolved.

### Statistical analyses

The χ^2^ test or Fisher’s exact was used to determine the significance of differences between covariates. Survival durations were calculated with the Kaplan–Meier method and analyzed by the log-rank test to compare cumulative survival durations among patient groups. The Cox proportional hazards model was used to compute univariate hazards ratios for the study parameters. In all of the tests, p-values <0.05 were defined as significant. The SPSS software program (SPSS Japan, Tokyo) was used for the analyses. MTT assay and wound-healing assay data are expressed as the mean ± standard deviation (SD), and differences in these data were analyzed using the unpaired Student’s t-test.

## Results

### CXCR2 expression on CCA cells

**Fig 1:**
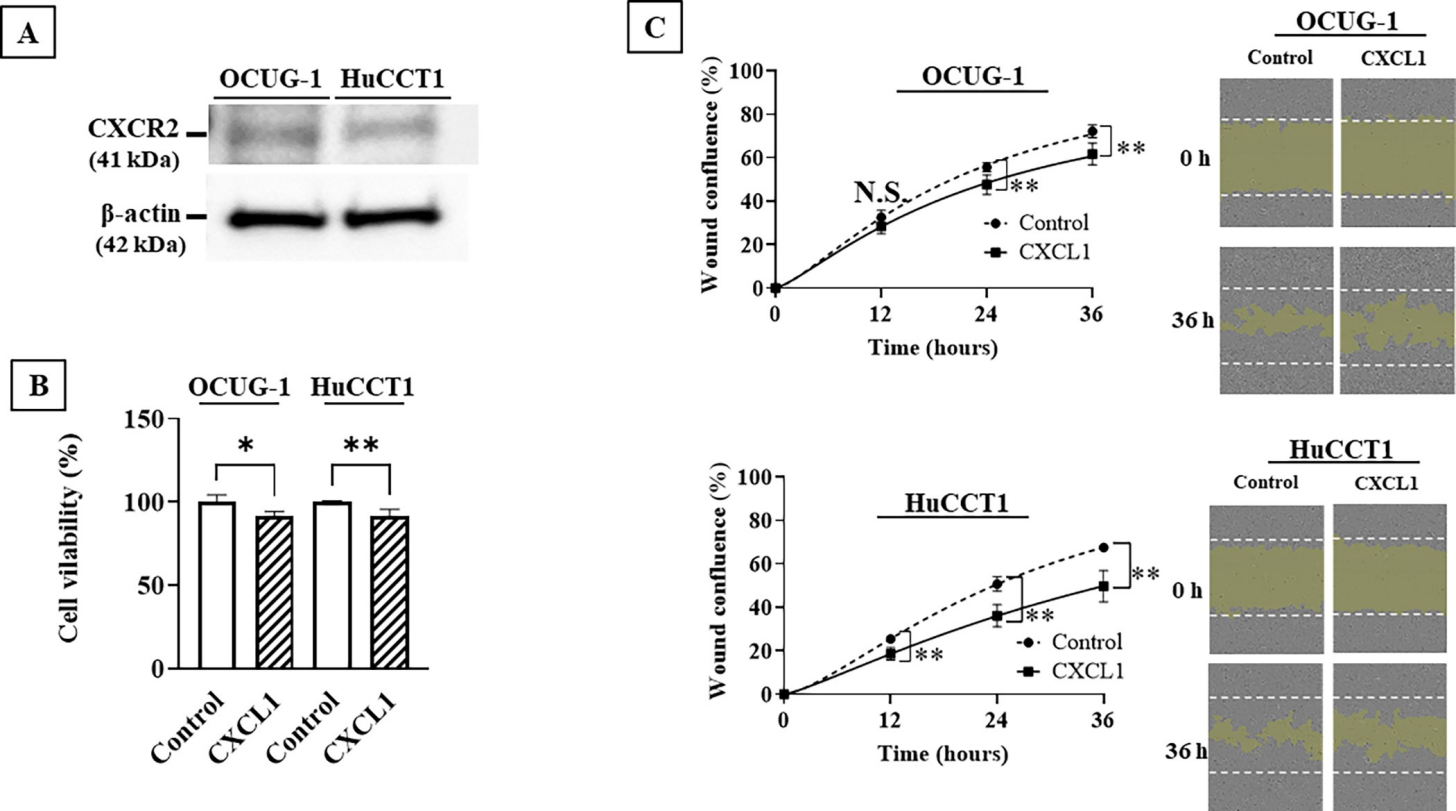
Effect of CXCL1 on the proliferation and migration of CCA cells. **A,** CXCR2 expression on OCUG-1 cells and HuCCT1 cells. **B,** CXCL1 significantly inhibited the proliferative potential of OCUG-1 cells and HuCCT1 cells at 72 h. **, p<0.01 vs control. **C,** CXCL1 significantly inhibited the migration potential of OCUG-1 cells after 24 h and HuCCT1 cells after 12 h. **, p<0.01 vs control.

**Fig 1A** provides an example of the CXCR2 expression level on both OCUG-1 cells and HuCCT1 cells are revealed by the western blotting analysis.

### Effects of CXCL1 on CCA cell proliferation and migration

CXCL1 decreased the proliferation of both OCUG-1 cells (p = 0.013) and HuCCT1 cells (p = 0.002) (**Fig 1B**) and significantly decreased the migration activities of OCUG-1 cells (p<0.001) and HuCCT1 cells (p = 0.01) **(Fig 1C**).

### Immunostaining of CXCL1 and CXCR2

**Fig 2:**
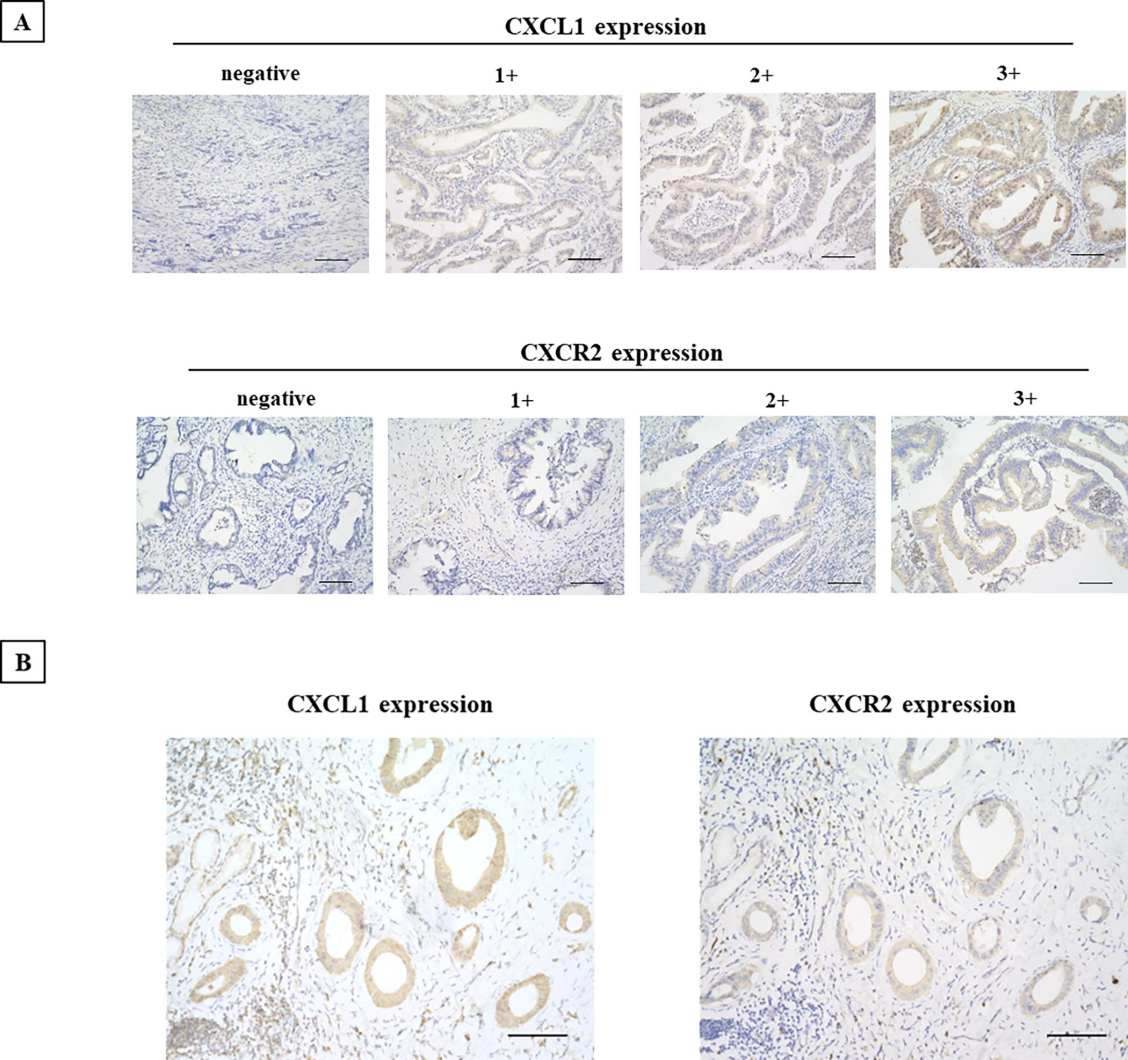
Representative immunostaining images of CXCL1 and CXCR2 expression. **A,** CXCL1 was expressed in CCA cells. CXCR2 was observed at both the cell membrane and the cytoplasm. Intensity score: 0, negative; 1+, weakly positive; 2+, positive; 3+. Bar: 100 μm. **B,** CXCL1 and CXCR2 expression in a case. Bar: 100 μm.

The results of the immunostaining study demonstrated that CXCL1 was expressed in the cytoplasm of CCA cells, and CXCR2 was expressed mainly at the cell membrane and the cytoplasm of the CCA cells (**Fig 2**). CXCL1 was positive in 130 of the 177 CCA cases (73.0%), and CXCR2 was positive in 32 cases (18.5%). Thirty-one cases (17.9%) were positive for both CXCL1 and CXCR2.

### The relationship between clinicopathological features and the expression of the CXCL1 and/or CXCR2

**Table 1** summarizes the correlations between the patients’ clinicopathologic features and the expression of CXCL1 or CXCR2 in their CCAs. The CXCL1 expression was significantly correlated with no distant metastasis (p = 0.043). The CXCR2 expression was not correlated with any clinicopathological features.

**Table 1:** The correlation between the clinicopathologic features and CXCL1 and/or CXCR2 in CCAs.

		CXCL1			CXCR2			CXCL1 and CXCR2		
Clinicopathological features		Positive (n = 130)	Negative (n = 48)	p-value	Positive (n = 32)	Negative (n = 141)	p-value	Positive (n = 31)	Negative (n = 142)	p-value
Age	≤70	63 (72.4%)	24 (27.6%)	0.865	16 (19.0%)	68 (81.0%)	0.692	16 (19.0%)	68 (81.0%)	0.843
	>70	67 (74.4%)	23 (25.6%)		16 (18.2%)	72 (81.8%)		15 (17.0%)	73 (83.0%)	
Gender	female	57 (75.0%)	19 (25.0%)	0.611	15 (20.8%)	57 (79.2%)	0.692	15 (20.8%)	57 (79.2%)	0.548
	male	70 (71.4%)	28 (28.6%)		17 (17.5%)	80 (82.5%)		16 (16.5%)	81 (83.5%)	
Histological type	well/mode	103 (75.2%)	34 (24.8%)	0.408	25 (18.9%)	107 (81.1%)	1	24 (18.2%)	108 (81.8%)	1
	por	12 (66.7%)	6 (33.3%)		3 (16.7%)	15 (83.3%)		3 (16.7%)	15 (83.3%)	
Stroma in tumors	med/int	75 (74.3%)	26 (25.7%)	0.212	19 (19.2%)	80 (80.8%)	0.347	19 (19.2%)	80 (80.8%)	0.347
	sci	4 (50.0%)	4 (50.0%)		0 (0.0%)	7 (100.0%)		0 (0.0%)	7 (100%)	
T invasion	T1	23 (79.3%)	6 (20.7%)	0.344	7 (25.0%)	21 (75.0%)	0.423	6 (21.4%)	22 (78.6%)	0.781
	T2-4	57 (67.9%)	27 (32.1%)		15 (18.1%)	68 (81.9%)		15 (18.1%)	68 (81.9%)	
Lymph node metastasis	Negative	83 (73.5%)	30 (26.5%)	1	21 (18.9%)	90 (81.8%)	1	20 (18.0%)	91 (82.0%)	1
	Positive	42 (72.4%)	16 (27.6%)		10 (17.9%)	46 (82.1%)		10 (17.9%)	46 (82.1%)	
Distant metastasis	Negative	112 (75.2%)	37 (24.8%)	0.043	28 (19.3%)	117 (80.7%)	1	27 (18.6%)	118 (81.4%)	1
	Positive	2 (33.3%)	4 (66.7%)		1 (16.7%)	5 (83.3%)		1 (16.7%)	5 (83.3%)	
Infiltration	Type A/B	99 (74.4%)	34 (25.6%)	0.514	26 (20.2%)	103 (79.8%)	0.217	25 (19.4%)	104 (80.6%)	0.213
	Type C	8 (66.7%)	4 (33.3%)		0 (0%)	11 (100.0%)		0 (0%)	11 (100%)	
Lymphatic invasion	Negative	51 (77.3%)	15 (22.7%)	0.344	13 (20.0%)	52 (80.0%)	1	12 (18.5%)	53 (81.5%)	1
	Positive	51 (69.9%)	22 (30.1%)		14 (19.7%)	57 (80.3%)		14 (19.7%)	57 (80.3%)	
Venous invasion	Negative	87 (76.3%)	27 (23.7%)	0.132	24 (21.2%)	89 (78.8%)	0.567	23 (20.4%)	90 (79.6%)	0.566
	Positive	15 (60.0%)	10 (40.0%)		3 (13.0%)	20 (87.0%)		3 (13.0%)	20 (87.0%)	
Stage	Stage 1	34 (73.9%)	12 (26.1%)	1	6 (13.0%)	40 (87.0%)	1	6 (13.0%)	40 (87.0%)	1
	Stage 2–4	60 (72.3%)	23 (27.7%)		12 (14.8%)	69 (85.2%)		12 (14.8%)	69 (85.2%)	

### CXCL1 and/or CXCR2 expression and the CCA patients’ survival

**Fig 3:**
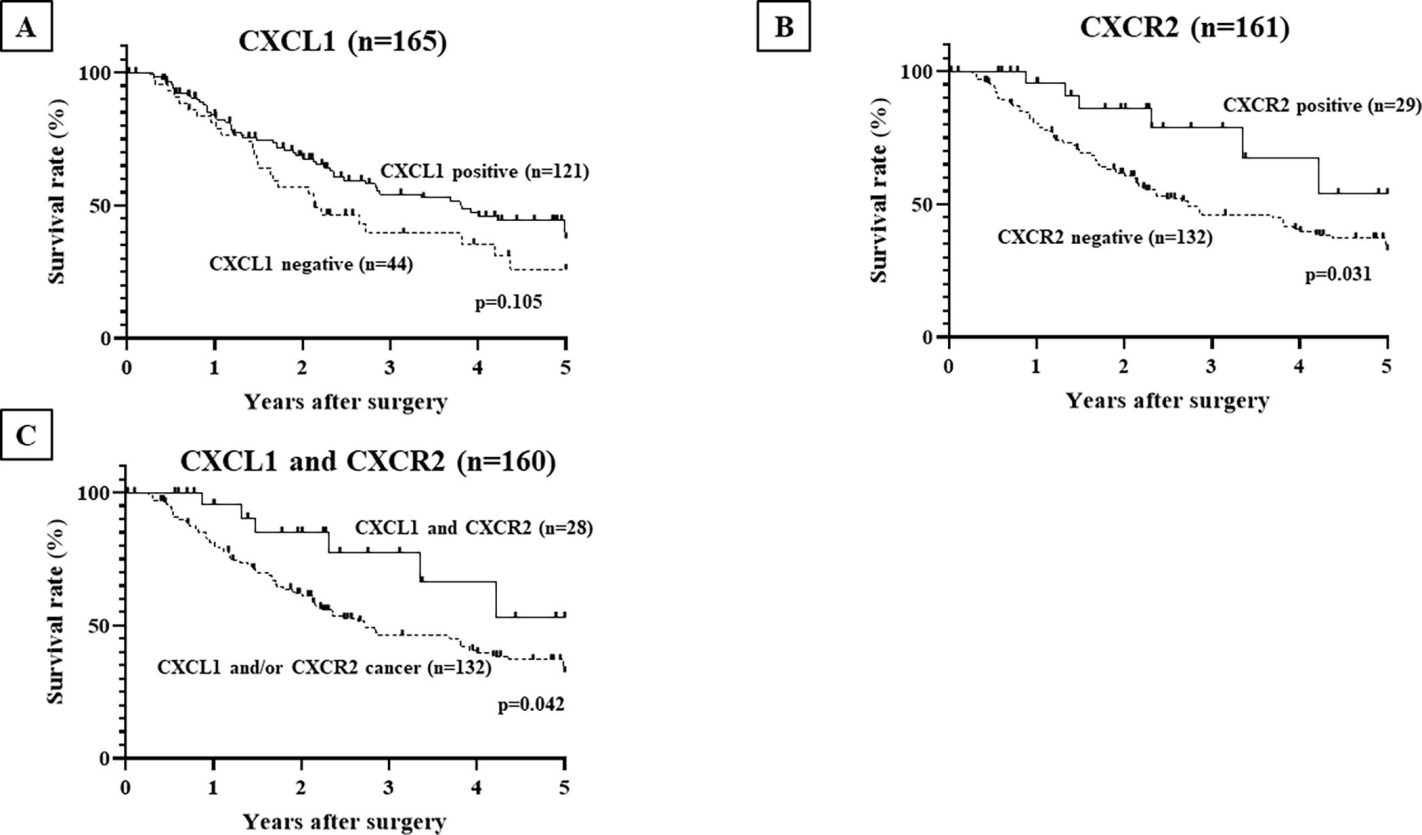
The overall survival rate of CCA patients according to CXCR2 expression. The patients with a positive expression of CXCR2 (n = 29) had a significantly better survival rate than those without CXCR2 expression (n = 132). The patients with positive expressions of both CXCL1 and CXCR2 (n = 28) had a significantly better survival rate than those with no CXCR2 expression (n = 132), but the patients with positive CXCL1 expression (n = 121) did not have a significantly better survival rate than those without CXCL1 expression (n = 44).

**Fig 3** provides the Kaplan-Meier survival curves for the CCA patients in terms of CXCL1 and/or CXCR2 expression. The overall survival of the CCA patients was not significantly different between the CXCL1-positive patients (n = 121) and the CXCL1-negative patients (n = 44) (**Fig 3A**). The CXCR2-positive patients (n = 29) had significantly better prognoses than the CXCR2-negative patients (n = 132) (p = 0.031) (**Fig 3B**). Patients with both CXCL1- and CXCR2-positive cancer (n = 28) had significantly better prognoses than those with CXCL1- and/or CXCR2-negative cancer (n = 132) (p = 0.042) (**Fig 3C**).

### Univariate and multivariate analyses of overall survival

The univariate analysis revealed that the overall survival of the patients was significantly correlated with CXCR2 expression in cancer cells, high age (≥70 years), T invasion (T2–T4), lymph node metastasis, and distant metastasis. The multivariate logistic regression analysis revealed that CXCR2 expression (p = 0.031) and lymph node metastasis (p = 0.004) were significantly correlated with the CCA patients’ overall survival (**Table 2**).

**Table 2:** Univariate and multivariate analysis with respect to overall survival.

Clinicopathologic features	Univariate			Multivariate		
	Hazard Ratio	95% CI	p-value	Hazard Ratio	95% CI	p-value
CXCL1	0.432–1.084	0.684	0.106			
CXCR2	0.177–0.933	0.406	0.034	0.139–0.910	0.356	0.031
CXCL1 and CXCR2	0.185–0.980	0.426	0.045			
Age ≥ 70	1.040–2.486	1.608	0.033	1.082–4.024	2.093	0.028
Gender	0.571–1.363	0.882	0.572	0.724–2.391	1.315	0.367
Histological type; por	0.793–2.849	1.503	0.212	0.424–2.292	0.987	0.973
T invasion	1.884–11.830	4.721	0.001	0.966–8.552	2.927	0.058
Lymph node metastasis	1.710–4.118	2.654	<0.001	1.337–4.596	2.481	0.004
Distant metastasis	1.279–8.147	3.228	0.013	0.795–19.704	3.927	0.093

## Discussion

We evaluated the significance of CXCL1 and CXCR2 signaling in CCA patients after surgical resection. CXCL1, one of the ligands of CXCR2, significantly decreased the proliferative and migratory potential of CCA cells. The immunohistochemical study also revealed an inverse correlation between CXCL1 and distant metastasis. CXCL1 may inhibit distant metastasis by suppressing the migratory potential of CCA cells. These results suggest that CXCL1 may act as a suppressor of CCA progression. Although other members of the CXCR2 ligand family, i.e., CXCL7 and CXCL5, have frequently been reported to promote the progression of CCA [1, 12, 13], our present results indicate that CXCL1 may exert a tumor-inhibitory effect on CCA cells. We considered that the reducing 80–90% viability in a 3-days might be clinically meaningful because the clinical effect is usually evaluated in a couple of months. It has been reported that the senescence of cancer cells is one of anti-proliferative responses against tumor progression via CXCL1-CXCR2 signaling, resulting in promoting apoptosis [23]. These mechanisms might be one of reasons for the tumor suppressive effect of CXCL1. These findings suggest that CXCL1-CXCR2 axis may play a tumor-suppressive role in the progression of CCA.

Since there has been no report about the clinicopathologic significance of CXCL1 or CXCR2 signaling in CCA, we next analyzed the correlation between the CXCR2 expression level and the clinicopathologic features in patients with CCA. The Kaplan-Meier curves demonstrated that the CXCR2-positive patients achieved significantly better survival, and the multivariate logistic regression analysis revealed that CXCR2 expression was significantly correlated with the overall survival of CCA patients. These findings suggested that CXCR2 might be a useful independent prognostic marker for CCA patients after surgical resection.

It has been reported that in triple-negative breast cancer, the expression of CXCR2 is associated with higher immune infiltration and more favorable outcomes [24], whereas CXCR2 ligands have been reported to have cancer-promoting effects [25–27]. Tumor progression-enhancing effects of CXCL7 and CXCL5 on CCA cells were described [1, 12, 13], in contrast, it has also been reported that CCL14 inhibited the proliferation and invasion of colon cancer cells [28]. Moreover, CCL14 attenuated the proliferation of hepatocellular carcinoma (HCC) cells by inhibiting the cell-cycle progression and promoting apoptosis, and it suppressed the growth via the Wnt/β-catenin signaling [23]. These findings may indicate that CXCL1-CXCR2 signaling exerts an inhibitory effect on the proliferative and migratory activity of CCA cells and thus improve the survival of patients with CCA.

In conclusion, the CXCL1-CXCR2 axis may play a tumor-suppressive role in the progression of CCA. CXCR2 might be a useful independent prognostic marker for CCA patients after surgical resection.

## Supporting information

S1 Raw images (PDF)

## References

[1] Caligiuri A, Pastore M, Lori G, Raggi C, Di Maira G, Marra F, et al. Role of Chemokines in the Biology of Cholangiocarcinoma. Cancers (Basel). 2020;12(8). doi: 10.3390/cancers12082215 32784743 PMC7463556

[2] Sarcognato S, Sacchi D, Fassan M, Fabris L, Cadamuro M, Zanus G, et al. Cholangiocarcinoma. Pathologica. 2021;113(3):158–69. doi: 10.32074/1591-951X-252 34294934 PMC8299326

[3] Macias RIR, Banales JM, Sangro B, Muntané J, Avila MA, Lozano E, et al. The search for novel diagnostic and prognostic biomarkers in cholangiocarcinoma. Biochim Biophys Acta Mol Basis Dis. 2018;1864(4 Pt B):1468–77. doi: 10.1016/j.bbadis.2017.08.002 28782657

[4] Macias RIR, Kornek M, Rodrigues PM, Paiva NA, Castro RE, Urban S, et al. Diagnostic and prognostic biomarkers in cholangiocarcinoma. Liver Int. 2019;39 Suppl 1:108–22. doi: 10.1111/liv.14090 30843325

[5] Rodrigues PM, Vogel A, Arrese M, Balderramo DC, Valle JW, Banales JM. Next-Generation Biomarkers for Cholangiocarcinoma. Cancers (Basel). 2021;13(13). doi: 10.3390/cancers13133222 34203269 PMC8269024

[6] Haga H, Yan IK, Takahashi K, Wood J, Zubair A, Patel T. Tumour cell-derived extracellular vesicles interact with mesenchymal stem cells to modulate the microenvironment and enhance cholangiocarcinoma growth. J Extracell Vesicles. 2015;4:24900. doi: 10.3402/jev.v4.24900 25557794 PMC4283029

[7] Ehling J, Tacke F. Role of chemokine pathways in hepatobiliary cancer. Cancer Lett. 2016;379(2):173–83. doi: 10.1016/j.canlet.2015.06.017 26123664

[8] Tanaka T, Bai Z, Srinoulprasert Y, Yang BG, Hayasaka H, Miyasaka M. Chemokines in tumor progression and metastasis. Cancer Sci. 2005;96(6):317–22. doi: 10.1111/j.1349-7006.2005.00059.x 15958053 PMC11158055

[9] Chow MT, Luster AD. Chemokines in cancer. Cancer Immunol Res. 2014;2(12):1125–31. doi: 10.1158/2326-6066.CIR-14-0160 25480554 PMC4258879

[10] Vandercappellen J, Van Damme J, Struyf S. The role of CXC chemokines and their receptors in cancer. Cancer Lett. 2008;267(2):226–44. doi: 10.1016/j.canlet.2008.04.050 18579287

[11] Bizzarri C, Beccari AR, Bertini R, Cavicchia MR, Giorgini S, Allegretti M. ELR+ CXC chemokines and their receptors (CXC chemokine receptor 1 and CXC chemokine receptor 2) as new therapeutic targets. Pharmacol Ther. 2006;112(1):139–49. doi: 10.1016/j.pharmthera.2006.04.002 16720046

[12] Guo Q, Jian Z, Jia B, Chang L. CXCL7 promotes proliferation and invasion of cholangiocarcinoma cells. Oncol Rep. 2017;37(2):1114–22. doi: 10.3892/or.2016.5312 27959418

[13] Okabe H, Beppu T, Ueda M, Hayashi H, Ishiko T, Masuda T, et al. Identification of CXCL5/ENA-78 as a factor involved in the interaction between cholangiocarcinoma cells and cancer-associated fibroblasts. Int J Cancer. 2012;131(10):2234–41. doi: 10.1002/ijc.27496 22337081

[14] Fang H, Li R, Gu Y, Fei Y, Jin K, Chen Y, et al. Intratumoral interleukin-9 delineates a distinct immunogenic class of gastric cancer patients with better prognosis and adjuvant chemotherapeutic response. Oncoimmunology. 2020;9(1):1856468. doi: 10.1080/2162402X.2020.1856468 33354409 PMC7738302

[15] Ohta M, Tanaka F, Yamaguchi H, Sadanaga N, Inoue H, Mori M. The high expression of Fractalkine results in a better prognosis for colorectal cancer patients. Int J Oncol. 2005;26(1):41–7. 15586223

[16] Hojo S, Koizumi K, Tsuneyama K, Arita Y, Cui Z, Shinohara K, et al. High-level expression of chemokine CXCL16 by tumor cells correlates with a good prognosis and increased tumor-infiltrating lymphocytes in colorectal cancer. Cancer Res. 2007;67(10):4725–31. doi: 10.1158/0008-5472.CAN-06-3424 17510400

[17] Wu Z, Huang X, Han X, Li Z, Zhu Q, Yan J, et al. The chemokine CXCL9 expression is associated with better prognosis for colorectal carcinoma patients. Biomed Pharmacother. 2016;78:8–13. doi: 10.1016/j.biopha.2015.12.021 26898419

[18] Minamiya Y, Saito H, Takahashi N, Ito M, Imai K, Ono T, et al. Expression of the chemokine receptor CXCR4 correlates with a favorable prognosis in patients with adenocarcinoma of the lung. Lung Cancer. 2010;68(3):466–71. doi: 10.1016/j.lungcan.2009.07.015 19716197

[19] Minamiya Y, Saito H, Takahashi N, Ito M, Toda H, Ono T, et al. Expression of the chemokine receptor CCR6 correlates with a favorable prognosis in patients with adenocarcinoma of the lung. Tumour Biol. 2011;32(1):197–202. doi: 10.1007/s13277-010-0113-x 20872189

[20] Li X, Sun S, Li N, Gao J, Yu J, Zhao J, et al. High Expression of CCR7 Predicts Lymph Node Metastasis and Good Prognosis in Triple Negative Breast Cancer. Cell Physiol Biochem. 2017;43(2):531–9. doi: 10.1159/000480526 28930757

[21] Yamamoto Y, Kuroda K, Sera T, Sugimoto A, Kushiyama S, Nishimura S, et al. The Clinicopathological Significance of the CXCR2 Ligands, CXCL1, CXCL2, CXCL3, CXCL5, CXCL6, CXCL7, and CXCL8 in Gastric Cancer. Anticancer Res. 2019;39(12):6645–52. doi: 10.21873/anticanres.13879 31810929

[22] Yamada N, Chung Y, Ohtani H, Ikeda T, Onoda N, Sawada T, et al. Establishment and characterization of a new human gallbladder carcinoma cell line (OCUG-1) producing TA-4. Int J Oncol. 1997;10(6):1251–5. doi: 10.3892/ijo.10.6.1251 21533512

[23] Do HTT, Lee CH, Cho J. Chemokines and their Receptors: Multifaceted Roles in Cancer Progression and Potential Value as Cancer Prognostic Markers. Cancers (Basel). 2020;12(2). doi: 10.3390/cancers12020287 31991604 PMC7072521

[24] Boissière-Michot F, Jacot W, Massol O, Mollevi C, Lazennec G. CXCR2 Levels Correlate with Immune Infiltration and a Better Prognosis of Triple-Negative Breast Cancers. Cancers (Basel). 2021;13(10). doi: 10.3390/cancers13102328 34066060 PMC8151934

[25] Luan J, Furuta Y, Du J, Richmond A. Developmental expression of two CXC chemokines, MIP-2 and KC, and their receptors. Cytokine. 2001;14(5):253–63. doi: 10.1006/cyto.2001.0882 11444905 PMC5433622

[26] Erin N, Tavşan E, Akdeniz Ö, Isca VMS, Rijo P. Rebound increases in chemokines by CXCR2 antagonist in breast cancer can be prevented by PKCδ and PKCε activators. Cytokine. 2021;142:155498. 33773907 10.1016/j.cyto.2021.155498

[27] Li C, Deng H, Zhou Y, Ye Y, Zhao S, Liang S, et al. Expression and clinical significance of CXC chemokines in the glioblastoma microenvironment. Life Sci. 2020;261:118486. doi: 10.1016/j.lfs.2020.118486 32976881

[28] Li N, Liang X, Li J, Zhang D, Li T, Guo Z. C-C motif chemokine ligand 14 inhibited colon cancer cell proliferation and invasion through suppressing M2 polarization of tumor-associated macrophages. Histol Histopathol. 2021:18348. 10.14670/HH-18-348 34096611

