# Supplementary material for: CXCR2 signaling might have a tumor-suppressive role in patients with cholangiocarcinoma
Source: PLoS One. 2022 Apr 4;17(4):e0266027. doi: 10.1371/journal.pone.0266027 (PMC8979434; doi:10.1371/journal.pone.0266027)
Supplement: S1 Raw images — (PDF) [file pone.0266027.s001.pdf]

CXCR2 (1:2000, R&D Systems, Minneapolis, MN)

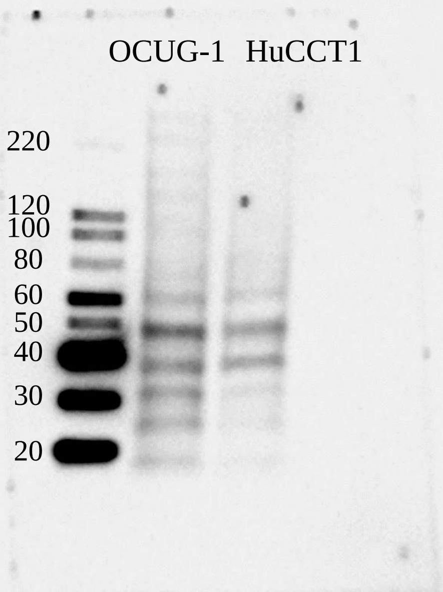

- Experimental sample
  - OCUG-1; 500ug/mL
  - HuCCT1; 500ug/mL
- Amount of sample use; 15uL/lane
- Gel; Mini-PROTEIN TGX Precast Gels 4-20% (Bio Rad)
- Electrophoresis conditions; 150V, 0.04A, 25min
- Chemiluminescence; ECL prime (GE Health Care)
- Detecting machine; FUSION SOLO. 7S
- Exposure time; 10min

β- actin (1:5000; Sigma-Aldrich)

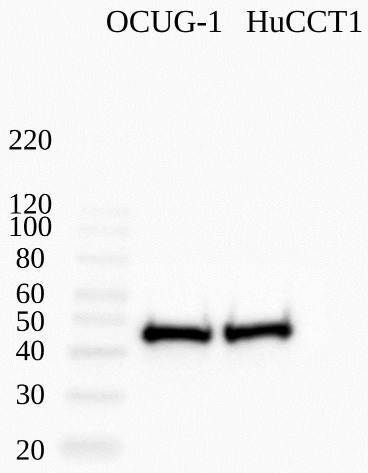

- Same membrane as on the left
- Stripping; Restore Western Blot Stripping Buffer (Thermo Fisher)
- Exposure time; 8.2sec
